# Effects of sensorimotor training on functional and pain outcomes in achilles tendinopathy: a systematic review

**DOI:** 10.3389/fspor.2024.1414633

**Published:** 2024-07-25

**Authors:** Myoung-Hwee Kim, Wille Martin, Andrew Quarmby, Josefine Stoll, Tilman Engel, Michael Cassel

**Affiliations:** University Outpatient Clinic, Sports Medicine & Sports Orthopaedics, University of Potsdam, Potsdam, Germany

**Keywords:** sensorimotor training, sensorimotor exercise, balance, stabilization, vibration, pain reduction, function enhancement, achilles tendinopathy management

## Abstract

**Background:**

Considering the neuromuscular alterations in Achilles tendinopathy (AT), sensorimotor training (SMT) might be beneficial to restore the neuromuscular capacity of the muscle-tendon complex and thereby improve patients' functions and alleviate symptoms. However, there is still a lack of knowledge concerning the effects of SMT on improving functional (e.g., strength) and pain outcomes in this population. Thus, the purpose of this study was to synthesize current evidence to analyze the efficacy of SMT in people with AT.

**Methods:**

A systematic electronic search was performed in PubMed, Web of Science, and Cochrane Central Register of Controlled Trials from inception to December 2023. Studies applying SMT in people with AT investigating functional or clinical pain outcomes were considered. Protocols had to incorporate balance, stabilization, proprioception, or vibration training. Patients with insertional or mid-portion AT (≥18 years age) diagnosed with clinical or sonographic evaluation were included.

**Results:**

The search yielded 823 records. A total of three randomized controlled trials were considered eligible for the analysis. Each trial used a different SMT protocol: balance training, balance with stabilization training, or whole-body vibration training (WBVT) with other co-interventions. Most functional and pain parameters improved compared to baseline. The first study reported a decrease in pain and an increase in performance (i.e., countermovement jump height) and endurance (i.e., number of heel-raises) by 12-week use of a balance training in addition to isometric, concentric/eccentric, and eccentric exercises. The second study evaluated the four weeks effect of SMT (balance and stabilization training plus eccentric exercises) in addition to passive physiotherapy (deep frictions, ice, ultrasound), resulting in an increased plantarflexion peak torque and reduced pain levels. The third study investigating WBVT reported at 12 weeks an increase in flexibility and a decrease in tendon pain.

**Discussion:**

SMT in addition to other co-interventions (i.e., eccentric, isometric, concentric/eccentric training, physiotherapy) showed improvements in strength, performance, muscle flexibility, and alleviated clinical outcomes of pain. SMT might therefore be useful as part of a multimodal treatment strategy protocol in patients suffering from AT. However, due to the small number of studies included and the diversity of SMT protocols, the current evidence is weak; its additional effectiveness should be evaluated.

**Systematic Review Registration:**

https://www.crd.york.ac.uk/PROSPERO/display_record.php?RecordID=467698, Identifier CRD42023467698.

## Introduction

Achilles tendinopathy (AT) is a chronic overuse condition that causes localized pain in the Achilles tendon with functional limitations when engaging in activities (1). It is problematic for both non-athletic and athletic populations. Epidemiological studies estimated a prevalence of 2.16 cases per 1,000 patient years in the general population and up to 36% in marathon runners (2). Tendinopathy has several anatomical (e.g., tenocytes) and biomechanical pain generators (e.g., mechanosensitive receptors and ion channels) that may be involved in nociception (3). In addition, recent research revealed the neuromuscular system associated with the condition (4). Altered muscle activity patterns of plantar flexors and a lowered temporal efficiency between muscle activation and mechanical force were reported in people with AT (4).

One of the most effective management strategies for AT has been recognized as eccentric calf exercise treatment, mostly conducted by use of the Alfredson loading protocol (5). It is theorized to increase tendon volume and tensile strength by influencing the production of type I collagen (6). Eccentric training may also reduce neovascularization and associated nerve ingrowth that accompanies the emergence of pain in some cases (7). Nevertheless, a previous systematic review suggested the effect of this training may be inferior to other heavy slow exercise interventions (8). Moreover, since the aim of the exercise treatment is to generate mechanical loading for improvement in muscle-tendon strength and endurance, the protocol may not address the issues of central motor impairment that were seen in AT (9). It was found that nearly 40% of people with AT still experience pain following the eccentric exercise protocol, meaning alternative interventions may be required for persistent cases (10).

Recently, Rio et al. focused on tendon neuroplastic training (TNT) to target the motor control impairments seen in AT (11). By use of TNT, isometric or isotonic strength training is combined with an externally paced audio or visual cue as opposed to self-paced exercise training. The authors suggested that TNT stimulates the neuromuscular system more effectively than traditional exercise treatments. This was supported by a mean increase of 22.25 points in Victorian Institute of Sport Assessment - Achilles questionnaire (VISA-A) scores after TNT, compared to 16.5 points after eccentric training. Additionally, 75% of participants in the TNT group and 58% in the eccentric training group exceeded the minimal clinically important difference (12). However, a recent meta-analysis showed little clinical evidence to support the use of TNT for reducing tendon pain over standard care (13).

Sensorimotor training (SMT), which was originally designed to restore physiological motor function in people suffering from chronic musculoskeletal clinical conditions, could be an effective alternative (14). SMT employs specific exercises to optimize the coordination and integration between the body's motor and sensory systems, aiming to enhance and optimize the sensorimotor system (15, 16). SMT is thought to affect and influence various parts of the sensorimotor system, with different terms used for specific descriptions (e.g., balance, proprioception, or vibration training). It places emphasis on postural control and introduces progressively challenging exercises to the sensorimotor system without relying on input from other sensory modalities like the TNT (11). SMT also focuses on postural control in a range of circumstances, eliciting automatic and reflexive muscle and active joint stabilization (16). A progressive balance training program that provokes automatic postural stabilization can be defined as an SMT protocol (16). In a similar sense, proprioception integrates sensory information, motor output, and brain processing for postural control as the primary somatosensory system component (17). Thus, proprioception training is defined as a specific training regimen that places emphasis on utilizing somatosensory signals, including proprioceptive or tactile afferents, in order to rehabilitate the sensorimotor system (15). Vibration training can be characterized as another kind of SMT that is specifically designed to stimulate motor neuron activity. The sensorimotor activity was shown to be increased during the vibration training compared to identical exercises without vibration (18), so the training regime is widely used for pathological conditions, especially for those who require sensorimotor alterations, like neurological disorders (19). Presently, such kinds of training are common in rehabilitation (20). Considering the sensorimotor alterations in AT (9), the usage of SMT might be beneficial to restore a normal sensorimotor capacity for muscles as well as for tendons. Nevertheless, no systematic analysis has been conducted to investigate the efficacy of SMT in an AT population.

Based on the current evidence, SMT could produce clinically meaningful improvements in the management of AT. Thus, the purpose of this study was to synthesize current evidence analyzing the outcomes of functional and pain parameters in AT following SMT (balance, stabilization, proprioception, or vibration training).

## Methods

### Data sources and search criteria

Relevant studies were searched in electronic databases, including PubMed, Web of Science, and the Cochrane Central Register of Controlled Trials, spanning from their inception to December 27, 2023. The search strategy involved combining three primary categories: intervention (exercise, training, therapy, rehabilitation, balance, stabilization, proprioception, vibration), pathology (tendinopathy, tendinitis, tendinosis, Achillodynia, paratenonitis, peritendinitis), and anatomical location (Achilles tendon, triceps surae, calcan*, plantarflex*) using the Boolean operator “OR” between the key terms and “AND” between the categories (Table 1 shows the search terms used in PubMed). Each search term was directed to the “title and abstract” headings, and filters such as article type (clinical trial, RCTs, etc.), language (English), and species (human) were applied if a database had the capability. Preferred Reporting Items for Systematic Reviews and Meta-Analyses (PRISMA) guidelines were followed for conducting and reporting the research (21). This study was pre-registered (PROSPERO: CRD42023467698).

**Table 1:** Search terms used in PubMed.

Combiners	Terms
Intervention	Exercise OR training OR therapy OR rehabilitation OR balance OR stabilization OR proprioception OR vibration
	AND
Anatomical location	Achilles tendon OR triceps surae OR calcan* OR plantarflex*
	AND
Pathology	Tendinopathy OR tendinitis OR tendinosis OR Achillodynia OR paratenonitis OR peritendinitis

### Eligibility criteria

Studies that prospectively investigated the effect of SMT on functional outcomes and clinical outcomes of pain in AT were considered. Eligible studies investigated people (≥18 years) with insertional or mid-portion AT diagnosed by clinical or sonographic evaluation. Studies were excluded if they were performed on healthy participants, children, adolescents, or animals. The functional outcomes included analysis of kinetic parameters such as strength (i.e., dorsiflexion and plantarflex peak torques), endurance (i.e., number of heel raises), and performance (i.e., jump height) or kinematic parameters such as range of motion (ROM) and muscle flexibility (i.e., passive resistive torque). For pain outcomes, the Visual Analogue Scale (VAS), VISA-A, or other measurement tools that are based on simple numerical rating scales (NRS) were considered. Studies were included if they measured a minimum of one of the outcome parameters whilst applying SMT in an AT population.

SMT was defined as a therapeutic training approach aimed at restoring normal motor function by employing specialized exercises, such as balance, stabilization, proprioception, or vibration training that optimize coordination and integration between the body's motor and sensory systems. An SMT protocol should be prescribed with specific guidelines (e.g., type, intensity, progression, and training period) that provoke the sensorimotor system through the Achilles tendon for at least four weeks. In each study, one of the groups had to implement a SMT protocol. Studies investigating the outcome parameters without groups undergoing specific or additional SMT programs were excluded. Co-interventions (e.g., eccentric training) with SMT were allowed. Included study designs were randomized controlled trials (RCTs).

### Study selection

After consolidating all the articles that were searched into a data sheet, two authors (MHK and WM) independently reviewed titles and abstracts to assess eligibility according to the inclusion and exclusion criteria. Subsequently, the remaining articles underwent full-text reviews to make the final decision. The corresponding authors were supposed to be contacted to obtain the full text if any articles were inaccessible, but this was not the case. Throughout the process, disagreements between the authors were resolved by discussion and consultation with a third author (MC), which was performed when consensus between the two authors was not reached.

### Methodological quality assessment

The Joanna Briggs Institute (JBI) quality assessment tools were used for the included studies. For each question, answers of “yes” or “no” were given. For “unclear” questions, the answer “no” was provided. The RCT scale consists of 13 criteria. Studies with scores of ≥9 were considered “high-quality” (equivalent to 70%), 6–8 were considered “medium-quality” (46%), and scores <6 were considered “low-quality” (39%). This JBI tool is widely used in the literature, establishing its relevance for use in systematic reviews (22, 23). Two reviewers (MHK and WM) assessed the methodological quality independently, and discrepancies were resolved by discussion.

### Data extraction

Data were extracted using a standardized data form [Microsoft Excel; version (2,303 build)]. Characteristics including study information (i.e., author, year, design), participant [i.e., sample size, sex, mean age (years), mean duration of symptoms (number of months), site of injury (insertion or mid-portion)], interventions (i.e., duration, type of intervention, sets & repetitions, frequency, progression), outcomes (name), results [mean ± standard deviation (SD), *p*-value], and the adherence of intervention were extracted. If there was no applicable mean and SD, other values such as median and interquartile range (IQR) were extracted. For studies that did not provide any data for pre and post comparisons, at least the description of results was taken. Disagreements between the authors (MHK and WM) were resolved by discussion.

### Data synthesis

The differences in the outcome measures before and after the interventions were described qualitatively because of the varied nature of the outcome parameters and the differences in exercise interventions.

Outcomes were categorized by task features (e.g., strength, performance) considering the SMT treatments applied. Where studies covered several relevant outcomes, the results from the single study were categorized accordingly. The levels of evidence (24) were provided to each outcome based on the methodological quality assessment with delta changes from the pre- to post-exercise as a percentage (%) where possible, to show the degree of changes in results.

## Results

### Study selection

**Figure 1:**
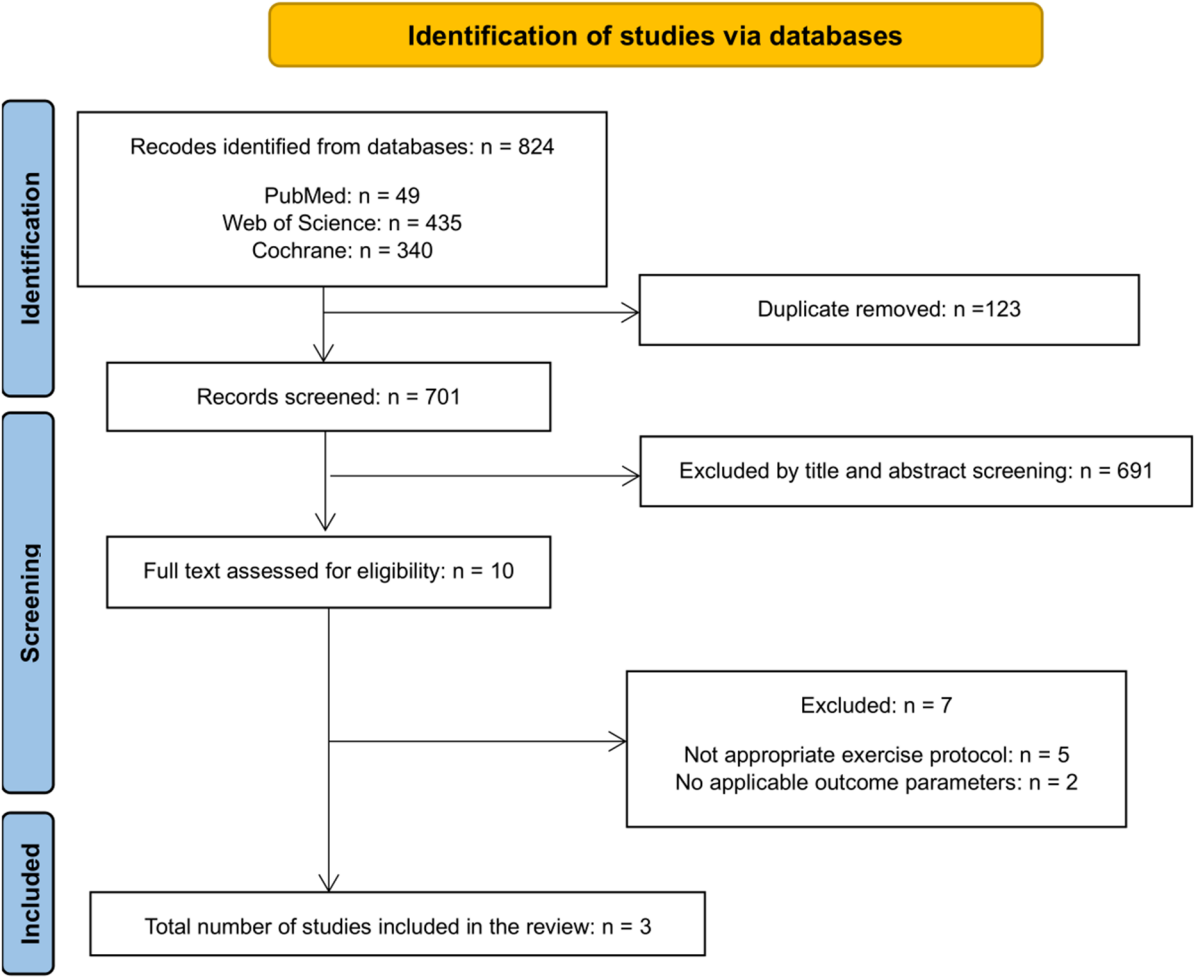
Study selection flowchart according to the PRISMA statements.

The search found a total of 824 studies, from which 112 duplicates were eliminated through automated title matching, with an additional 11 duplicates confirmed manually using Microsoft Excel [version (2,303 build)]. Subsequently, 691 articles were excluded during the screening process of titles and abstracts. The remaining 10 articles underwent a comprehensive full-text eligibility assessment. Among them, 5 studies were excluded due to inappropriate exercise interventions (e.g., eccentric and concentric combined exercise treatment), while 2 studies lacked relevant outcome parameters (e.g., rectus abdominis thickness), leaving 3 applicable studies (Figure 1). Detailed information on the included and excluded studies is shown in the Supplementary File S2.

### Methodological quality assessment

A total of three RCT studies were included (25–27). The average quality score on the RCT scale was 8 points, with individual scores of 7, 8, and 10 points for each study, respectively. One study was ranked as high-quality (25), and two studies were raked as medium-quality (26, 27). Subject allocation was concealed by one study (25). All three studies performed blinding of assessors, but only one study met the criteria of blinding therapists (25). Blinding of subjects was marked “no” in all the studies due to the nature of exercise treatments. Two studies adequately described the complete follow-up (26, 27), and two studies retained the same number of subjects until the post-measurement (25, 26). However, only one study was marked as conducting appropriate statistical analysis (25) because the other studies did not consider a power analysis (26, 27). The detailed contents of the quality assessment are seen in Table 2.

**Table 2:** Joanna briggs institute (JBI) randomized controlled trial studies tool.

Study (year)	1	2	3	4	5	6	7	8	9	10	11	12	13	Total score	Quality assessment
Silbernagel et al. (27)	Y	N	Y	N	N	Y	Y	Y	N	Y	Y	N	Y	8	Medium
Mayer et al. (26)	N	N	Y	N	N	Y	Y	Y	Y	Y	N	N	Y	7	Medium
Horstmann et al. (25)	Y	Y	Y	N	Y	Y	Y	N	Y	Y	N	Y	Y	10	High

1. Was true randomization used for assignment of participants to treatment groups?

2. Was allocation to treatment groups concealed?

3. Were treatment groups similar at the baseline?

4. Were participants blind to treatment assignment?

5. Were those delivering treatment blind to treatment assignment?

6. Were outcomes assessors blind to treatment assignment?

7. Were treatment groups treated identically other than the intervention of interest?

8. Was follow up complete and if not, were differences between groups in terms of their follow up adequately described and analyzed?

9. Were participants analyzed in the groups to which they were randomized?

10. Were outcomes measured in the same way for treatment groups?

11. Were outcomes measured in a reliable way?

12. Was appropriate statistical analysis used?

13. Was the trial design appropriate, and any deviations from the standard RCT design (individual randomization, parallel groups) accounted for in the conduct and analysis of the trial?

### Characteristics of included studies

The total number of subjects was 126, comprised of 35 (27.8%) females and 63 (50%) males, with 28 (22.2%) unknown cases. Among that, the subjects of SMT groups were 56, comprised of 15 (26.8%) females and 30 (53.6%) males, with 11 (19.6%) unknown sexes. While the mean age of all subjects was 42.3 years, ranging from 41 ± 5.9 years (26) to 47 ± 14.7 years (27), the mean age of the SMT subjects was 45 years, ranging from 35 ± 6.7 years (26) to 47 ± 14.7 years (27). The mean duration of symptoms was 20 months, ranging from 7.9 ± 6.8 months (26) to 41 ± 55.9 (27) months, and the mean duration of symptoms in SMT subjects was 18.7 months, ranging from 17.3 ± 18.7 (26) to 20 ± 25.4 (27) months. One study did not state the symptom duration (25). Two studies included mid-portion AT (26, 27), whereas one study included insertion and mid-portion AT (25). The characteristics of the included studies are summarized in Table 3.

**Table 3:** Characteristics of the included studies.

Study (year)	Deign	Intervention	Sample size (Female/male)	Mean age in years	Symptom duration in months	Outcomes	Results
Silbernagel et al. (27)	RCT	A:Silbernagel protocol B: CT	22 (5/17) 18 (4/14)	47 ± 14.7 41 ± 10.2	20 ± 25.4 41 ± 55.9	CMJ height (cm)	**A**; 13 ± 7 vs. 14 ± 7.9 (*P* < 0.05, +8%), **B**; 6 vs. 16 ± 2.9 (*P* < 0.05, +6.7%), Significantly increased in both groups
						Plantarflexion ROM (degrees)	**A**; 72 ± 6.9 vs. 73 ± 5 (+1%), **B**; 73 ± 6.6 vs. 72 ± 5.7 (−1.4%)
						Toe-raise test (numbers)	**A**; 20 ± 11 vs. 24 ± 10.8 (*P* < 0.05, +20%), **B**; 22 ± 11 vs. 28 ± 14.9 (*P* < 0.05, +27.3%), Significantly increased in both groups
						VAS (palpation pain)	**A**; 49 ± 26.2^a^ vs. 35 ± 24.8^a^ (*P* < 0.05, −29%), **B**; 27 ± 21.5^a^ vs. 31 ± 26^a^ (+14.8%)
Mayer et al. (26)	RCT	A: Physiotherapy intervention (sensory motor training + deep- friction massage + local pulsed ultrasound + ice) B: Custom insole C: Control	11 (sex not stated) 9 (sex not stated) 8 (sex not stated)	41 ± 5.9 35 ± 6.7 38 ± 4.9	17.3 ± 18.7 13.8 ± 16.5 7.9 ± 6.8	Dorsiflexion peak torque	**A, B, C**; not increased (mean <10%)
						Plantarflex peak torque	**A, B**; Significantly increased (mean >10%), **C**; not increased (mean <10%)
						Pain Disability Index (PDI) during activities of daily life	**A, B**; Significantly Improved (*P* < 0.05), **C**; not improved
						Pain Experience Scale (PES) at pre-test, after treadmill run and strength test	**A, B**; Pain was increased up to a maximum following the tests before the intervention, whereas pain was not increased after the interventions, **C**; Pain was increased up to a maximum following the tests before and after the intervention
Horstmann et al. (25)	RCT	A: Whole-body vibration training B: ET C: Wait-and-see approach	23 (10/13) 19 (9/10) 16 (7/9)	46 ± 6.9 45.7 ± 8.5 44.4 ± 7.7	Not stated Not stated Not stated	Passive resistive torques (Nm) at 0°, 5°, 10°, 15°, 20°, and 25° during ankle dorsiflexion	**A**; Significantly increased at 10°, 15°, 20°, and 25° angles (*P *< 0.05), **B**; Significantly increased at 15°, 20°, and 25° angles (*P *< 0.05), **C**; Not increased
						Concentric plantarflexion and dorsiflexion assessment at 60°/s	**A**; Increased during plantarflexion, **B**; Increased during plantarflexion and dorsiflexion **C**; Not increased
						Concentric/eccentric plantarflexion and dorsiflexion assessment at 20°/s	**A**; Increased during concentric plantarflexion, **B**; Increased during concentric/eccentric plantarflexion and dorsiflexion **C**; Not increased
						VAS (palpation pain at osseous insertion, 2 cm proximal to insertion, and at musculotendinous junction)	**A**; Significantly improved at osseous insertion; 25.4 ± 32.3 vs. 10.7 ± 22.8 (*P* < 0.05, −57.9%), at 2 cm proximal to insertion; 72.9 ± 31.4 vs. 35.4 ± 32.1 (*P *< 0.05, −51%), but not at musculotendinous junction; 33.6 ± 31.4 vs. 34.4 ± 34.2 (+2.4%), **B**; Significantly improved at osseous insertion; 28.4 ± 31.9 vs. 5.6 ± 15.0 (*P* < 0.05, −80.3%), at 2 cm proximal to insertion; 69.0 ± 33.6 vs. 22.6 ± 27.8 (*P *< 0.05, −67.3%), and at musculotendinous junction; 47.0 ± 36.1 vs. 16.4 ± 24.1 (*P *< 0.05, −65.1%), **C**; Significantly improved at osseous insertion; 23.3 ± 30.2 vs. 11.9 ± 20.1 (*P* < 0.05, −48.9%), at 2 cm proximal to insertion; 62.6 ± 28.5 vs. 45.1 ± 31.4 (*P *< 0.05, −28%), but not at musculotendinous junction; 18.7 ± 24.3 vs. 39.3 ± 31.4 (*P *< 0.05, +110.2%)
						VAS (impact of pain on recreation, running training, other physical activities, family responsibility at home, and social activities)	**A**; Significantly improved during recreation; 27.2 ± 27.3 vs. 15.8 ± 21.3 (*P *< 0.05, −41.9%), running training; 60.2 ± 35.0 vs. 35.3 ± 34.7 (*P *< 0.05, −41.4%), other physical activities; 45.3 ± 35.0 vs. 24.4 ± 27.7 (*P *< 0.05, −46%), but not during family responsibility at home; 8.4 ± 19.7 vs. 3.8 ± 6.2 (−54.8%), and social activities; 5.8 ± 12.9 vs. 3.3 ± 7.0 (−43%), **B**; Significantly improved during recreation; 29.1 ± 23.0 vs. 9.4 ± 16.9 (*P *< 0.05, −67.7%), running training; 76.3 ± 27.3 vs. 24.7 ± 30.3 (*P *< 0.05, −64.1%), other physical activities; 54.1 ± 31.4 vs. 14.2 ± 21.5 (*P *< 0.05, −73.8%), family responsibility at home; 13.0 ± 17.7 vs. 5.5 ± 15.8 (*P *< 0.05, −57.7%), and social activities; 9.4 ± 15.8 vs. 1.0 ± 2.0 (*P *< 0.05, −89.4%), **C**; Improved but not significant during recreation; 29.7 ± 30.3 vs. 19.8 ± 26.2 (−33.3%), running training; 63.9 ± 33.6 vs. 51.0 ± 38.1 (−20.2%), other physical activities; 40.0 ± 31.5 vs. 27.2 ± 33.8 (−32%), family responsibility at home; 16.0 ± 28.2 vs. 10.2 ± 26.5 (−36.3%), and not improved during social activities; 7.2 ± 17.1 vs. 9.9 ± 16.9 (+38%)

Data is presented in mean ± standard deviation.

CT, concentric training; ET, eccentric training; RCT, randomized controlled trial; CCT, controlled clinical trial; CMJ, counter movement jump; VAS, visual analogue scale; VISA-A, Victorian Institute of Sports Assessment – Achilles; NRS, numerical rating scale.

^a^ Median ± Inter Quartile Range.

### Characteristics of the interventions

The first study by Silbernagel et al. compared a group performed the Silbernagel protocol (balance training with isometric, concentric, and eccentric training) to a group that underwent concentric training for 12 weeks (27). The second study by Mayer et al. compared a group undergoing physiotherapy (balance and stabilization training combined with deep-friction massage, local pulsed ultrasound, and icing) to a second group wearing custom fitted insoles and a third group of untreated controls after a period of 4 weeks (26). The third study by Horstmann et al. compared a group that performed WBVT (with intermittent concentric and eccentric loadings) to a second group performing eccentric training and a third group of controls who maintained their recreational activities for 12 weeks (25). A detailed overview of the interventions is summarized in the Supplementary File S3.

### Changes of functional outcomes

#### Strength, performance, and range of motion

The study by Mayer et al. using balance plus stabilization training showed a significant increase in plantarflexion peak torque at 4 weeks (26), whereas the study by Horstmann et al. examining WBVT reported no significant increases in concentric and eccentric plantarflexion peak torques at 20°/s and 60°/s after 12 weeks (25). Regarding dorsiflexion, both studies showed no significant increases (25, 26). The study by Silbernagel et al. using balance training showed significant increases in countermovement jump (+8%) and in the number of toe-raises (+20%) at 12 weeks (27). Conversely, no significant changes in plantarflexion ROM (+1%) were seen (27).

### Changes of pain outcomes

Balance training led to a significant decrease in VAS measured at the most painful site (−29%) at 12 weeks (27). The study with WBVT reported significant decreases at osseous insertion (−57.9%) and 2 cm proximal to insertion (−51%), but not at musculotendinous junction (+2.4%) at 12 weeks (25). Pain relief was found to be present during daily activities by use of balance plus stabilization training and WBVT at 4 and 12 weeks, respectively (25, 26). In addition, Mayer et al. reported no increase in pain index after a treadmill run and a strength test (26).

## Discussion

The purpose of this systematic review was to synthesize and appraise the results of SMT (i.e., balance, stabilization, proprioception, or vibration training) on functional and clinical outcomes of pain in people with AT. The search yielded three eligible studies investigating the effects of SMT as a co-intervention to high loading strategies (e.g., in addition to eccentric training). Conflicting results were observed for strength outcomes on plantar flexion torque, with positive short-term effects following balance plus stabilization training and no effects after WBVT. Improvements in performance outcomes (countermovement jump and number of toe-raises) were found in one study, while there were no relevant effects on plantarflexion ROM detectable. All studies reported reduced clinical pain outcomes following different SMT regimens.

### Functional outcomes of SMT

This review indicates improvements in performance and strength outcomes following different SMT regimens, while ROM was unaffected. Specifically, in terms of strength outcomes, one study that applied balance plus stabilization training showed a significant increase in plantarflexion peak torque (26), while another study using the WBVT reported insignificant increases in concentric and eccentric plantarflexion peak torques at 20°/s and 60°/s (25).

In the study of Mayer et al., significant increases in strength measured by plantar flexor peak torque and pain during activities of daily living at four weeks when applying balance and stabilization exercises (physiotherapy group) has been reported (26). The extent to which the SMT is responsible for the positive short-time outcome stays unclear. Interestingly, the insole group showed similar improvements like the physiotherapy group. The authors explained the improvements by changes in neuromuscular control due to modulation of afferent input to influence neuromuscular regulation (i.e., reduced spinal inhibition) leading to an optimized dynamic joint stability and postural control, respectively (26). Clinical effectiveness of insoles is also supposed to develop by an early optimization of muscular-regulated joint stability leading to pain-relief and increased strength outcomes. Exemplarily, a combination of longitudinal arch support with rear foot stabilization led directly (compression of the peroneal tendon) or indirectly (stimulation of the proprioceptors by altering joint position) to modulation of the afferent input (28). The fact that the insole effects can be expected to be almost completed after 4 weeks (28) supports this hypothesis since it is well known that sensorimotor training effects are present within 4 weeks (29). However, the design of the study of Mayer et al. did not allow for clarification of the effect mechanism of insoles (26).

Considering the study of Silbernagel et al. improvements observed after balance plus stabilization training could be attributed to the inclusion of drop jumps and countermovement jumps, which involve plantarflexion movements (27). Conversely, the WBVT study included only a few intermittent heel-raises (25). The insignificant increases in dorsiflexion peak torque in both studies can be explained by the influence of contraction mode on training gains (30).

The outcome parameter of plantarflexion ROM showed an insignificant increase (+1%) (27). However, increased ankle ROM has been reported to be clinically relevant (31). This might be due to increased tendon compliance, potentially indicating reduced loading capacity (31). Interestingly, a previous systematic review did not recommend plantarflexion and dorsiflexion ROM as reliable outcome measures in the AT population (23). Therefore, the ankle ROM has to be interpreted with caution in general.

In the study reporting improvements in performance (countermovement jumps and number of toe- raises), participants engaged in balance training, which included 30 s of one-leg standing for 5 sets, and 5 m of toe or heel walking for 5 sets, performed from three times a day to once a day (27). To maintain balance, the neuromuscular system collaborates with the somatosensory, visual, and vestibular systems to control the body and stabilize the body's center of mass. This cooperative sensory information forms the basis of the ability to control balance (32). Thus, this type of training potentially creates new response strategies (33) by modulation of afferent input (29) that may lead to improvements in functional outcome parameters.

### Pain outcomes of SMT

Significant decreases were noted at most measurement sites, including the osseous insertion and 2 cm proximal to the insertion of the Achilles tendon after WBVT (25), and at the most painful site following balance training (27). These findings underscore the potential effectiveness of additional SMT regimens in reducing pain associated with AT. However, pain at the musculotendinous junction (MTJ) did not decrease following the vibration training (25). The authors speculated that this discrepancy may be due to the unique characteristics of the MTJ (25). This region may possess histological or biomechanical properties that make it more susceptible to the effects of vibration (25). The MTJ is a critical transition zone between muscle and tendon, experiencing high mechanical stress and strain during intense activities (34). Its unique morphology, characterized by a highly folded muscle membrane infiltrated with collagen fibrils from the tendon (35), further complicates its response to vibration training. Consequently, it may be less responsive to the mechanical stimuli provided by the vibration training (36). These multifaceted factors warrant further investigation to elucidate the underlying reasons.

Pain relief is largely influenced by rapid neural changes, while functional improvements potentially require long-term physiological adaptations lasting up to six months, influenced not only by neural systems but also by various factors such as muscular systems and tendon structure (33). SMT enhances proprioception, the body's ability to sense position and movement, leading to improved coordination and postural control, which may reduce strain on the Achilles tendon during activities, thereby alleviating pain (37). In contrast, functional improvements result from long-term physiological adaptations (38) involving complex biological processes like tendon structure reorganization, and adjustments in neural and muscular systems (33).

Future studies ought to extend the duration of interventions to capture long-term physiological adaptations and include comprehensive assessments of both neural and physiological changes. Additionally, researchers should ensure larger sample sizes and standardized protocols to enhance the generalizability and consistency of findings across various treatments.

### Limitations

There are limitations that should be considered. Two out of three studies did not conceal allocation to treatment groups, which might cause selection bias (26, 27). Also, the same two studies failed to blind therapists who administered the training intervention, so performance bias might have been present (26, 27). Moreover, data for training adherence was reported only in one study that implemented vibration training (25), so it was impossible to draw a concrete conclusion regarding the effect of balance and balance plus stabilization exercises that were conducted in the other two studies (26, 27). Furthermore, due to the lack of available studies that implemented SMT, its effectiveness to eccentric training was not investigated. In addition, the small number of studies included in this review and the variability among the interventions can hinder consistent conclusions and limit the generalizability of the findings.

Although eccentric exercise is recognized in literature as the gold standard conservative management for the population of AT, it is questionable whether the effect is different from other exercise therapies (39). According to the “time-under-tension” hypothesis (40), beneficial adaptations can occur with any sort of loading as long as the mechanical strain is applied adequately within the optimal range of 4.5%–6.5% (41, 42). Recently, the personalized isometric training interventions that addressed muscle-tendon imbalances showed improvements in the outcomes of the triceps surae and knee extensor muscle strength (41–43). The authors argued that muscle and tendon tissues exhibit distinct sensitivities to mechano-metabolic stimuli, with muscle adaptation being responsive to various metabolic stress, while tendon adaptation is predominantly driven by the experienced strain (43). This differential adaptation can lead to significant imbalances, causing high level tendon strain that is closely associated with tendinopathies (34, 44). The results of the studies emphasize individualized training regimens considering the balance between muscle and tendon development to mitigate injury risk (41–43). However, its effectiveness in single treatment or combined treatment modalities has to be explored.

### Clinical implication

The main finding of the present systematic review is that SMT might be considered as an optional exercise treatment in addition to other co-interventions, such as eccentric training, for AT population. Clinicians may consider adding balance with stabilization components while performing eccentric training, e.g., by use of a stability pad. One-leg balance for 5 sets of 30 s and toe or heel walking for 5 sets of 5 m could be the examples as seen in the Silbernagel et al. study. Those exercises are applicable to a wide range of patients with co-morbidities and do not require much time, with a duration of approximately 10 min, including rest in between sessions. Therein implementation as co-interventions might bring meaningful results as early as 4 weeks when additionally performed to standard care two or three times a week.

## Conclusion

This is the first study systematically investigating the efficacy of SMT on functional and clinical outcomes of pain in people with AT, identifying an area that needs further exploration. The search yielded three eligible studies investigating the effects of SMT as a co-intervention to high loading strategies (e.g., in addition to eccentric training). SMT, in addition to other interventions (e.g., eccentric training, physiotherapy), showed potential effects on strength outcomes in short-term and improvements in performance outcomes (i.e., countermovement jump and number of toe-raises). In addition, all the included studies with different SMT regimens reported reduced clinical pain outcomes. SMT can therefore be recommended as part of a multimodal treatment strategy protocol in patients suffering from Achilles tendinopathy. However, the current evidence is weak; its additional effectiveness to golden standard therapy (high loading protocols) should be evaluated. The small number of studies included in this review and the variability between the SMT protocols impedes the ability to draw consistent conclusions across study protocols including SMT loads and modalities used. Future studies ought to extend the duration of interventions to capture long-term physiological adaptations and include comprehensive assessments of both neural and physiological changes. Additionally, researchers should ensure larger sample sizes and standardized protocols to enhance the generalizability and consistency of findings across various treatments.

## Data Availability

The original contributions presented in the study are included in the article/**Supplementary Material**, further inquiries can be directed to the corresponding author.
