# Supplementary material for: Effects of sensorimotor training on functional and pain outcomes in achilles tendinopathy: a systematic review
Source: Front Sports Act Living. 2024 Jul 25;6:1414633. doi: 10.3389/fspor.2024.1414633 (PMC11306088; doi:10.3389/fspor.2024.1414633)
Supplement: Supplementary file 1 [file Table1.docx]

**Supplemental File 1 – PRISMA 2020 Checklist**

| **Section and Topic** | **Item #** | **Checklist item** | **Location where item is reported** |
| --- | --- | --- | --- |
| **TITLE** | | |  |
| Title | 1 | Identify the report as a systematic review. | **☑** P 1 |
| **ABSTRACT** | | |  |
| Abstract | 2 | See the PRISMA 2020 for Abstracts checklist. | **☑** P 1 |
| **INTRODUCTION** | | |  |
| Rationale | 3 | Describe the rationale for the review in the context of existing knowledge. | **☑** P 3 |
| Objectives | 4 | Provide an explicit statement of the objective(s) or question(s) the review addresses. | **☑** P 3 - 4 |
| **METHODS** | | |  |
| Eligibility criteria | 5 | Specify the inclusion and exclusion criteria for the review and how studies were grouped for the syntheses. | **☑** P 4 |
| Information sources | 6 | Specify all databases, registers, websites, organisations, reference lists and other sources searched or consulted to identify studies. Specify the date when each source was last searched or consulted. | **☑** P 4 |
| Search strategy | 7 | Present the full search strategies for all databases, registers and websites, including any filters and limits used. | **☑** P 4 |
| Selection process | 8 | Specify the methods used to decide whether a study met the inclusion criteria of the review, including how many reviewers screened each record and each report retrieved, whether they worked independently, and if applicable, details of automation tools used in the process. | **☑** P 4 |
| Data collection process | 9 | Specify the methods used to collect data from reports, including how many reviewers collected data from each report, whether they worked independently, any processes for obtaining or confirming data from study investigators, and if applicable, details of automation tools used in the process. | **☑** P 5 |
| Data items | 10a | List and define all outcomes for which data were sought. Specify whether all results that were compatible with each outcome domain in each study were sought (e.g. for all measures, time points, analyses), and if not, the methods used to decide which results to collect. | **☑** P 5 |
|  | 10b | List and define all other variables for which data were sought (e.g. participant and intervention characteristics, funding sources). Describe any assumptions made about any missing or unclear information. | **☑** P 5 |
| Study risk of bias assessment | 11 | Specify the methods used to assess risk of bias in the included studies, including details of the tool(s) used, how many reviewers assessed each study and whether they worked independently, and if applicable, details of automation tools used in the process. | **☑** P 5 |
| Effect measures | 12 | Specify for each outcome the effect measure(s) (e.g. risk ratio, mean difference) used in the synthesis or presentation of results. | **☑** P 5 |
| Synthesis methods | 13a | Describe the processes used to decide which studies were eligible for each synthesis (e.g. tabulating the study intervention characteristics and comparing against the planned groups for each synthesis (item #5)). | **☑** P 5 |
|  | 13b | Describe any methods required to prepare the data for presentation or synthesis, such as handling of missing summary statistics, or data conversions. | **☑** P 5 |
|  | 13c | Describe any methods used to tabulate or visually display results of individual studies and syntheses. | **☑** P 5 |
|  | 13d | Describe any methods used to synthesize results and provide a rationale for the choice(s). If meta-analysis was performed, describe the model(s), method(s) to identify the presence and extent of statistical heterogeneity, and software package(s) used. | **☑** P 5 |
|  | 13e | Describe any methods used to explore possible causes of heterogeneity among study results (e.g., subgroup analysis, meta-regression). | **☑**Not applicable |
|  | 13f | Describe any sensitivity analyses conducted to assess robustness of the synthesized results. | **☑**Not applicable |
| Reporting bias assessment | 14 | Describe any methods used to assess risk of bias due to missing results in a synthesis (arising from reporting biases). | **☑** Not applicable |
| Certainty assessment | 15 | Describe any methods used to assess certainty (or confidence) in the body of evidence for an outcome. | **☑**Not applicable |
| **RESULTS** | | |  |
| Study selection | 16a | Describe the results of the search and selection process, from the number of records identified in the search to the number of studies included in the review, ideally using a flow diagram. | **☑** P 5 |
|  | 16b | Cite studies that might appear to meet the inclusion criteria, but which were excluded, and explain why they were excluded. | **☑** P 5 |
| Study characteristics | 17 | Cite each included study and present its characteristics. | **☑** P 6 |
| Risk of bias in studies | 18 | Present assessments of risk of bias for each included study. | **☑** P 5 |
| Results of individual studies | 19 | For all outcomes, present, for each study: (a) summary statistics for each group (where appropriate) and (b) an effect estimate and its precision (e.g. confidence/credible interval), ideally using structured tables or plots. | **☑** P 6 - 7 |
| Results of syntheses | 20a | For each synthesis, briefly summarise the characteristics and risk of bias among contributing studies. | **☑** P 6 - 7 |
|  | 20b | Present results of all statistical syntheses conducted. If meta-analysis was done, present for each the summary estimate and its precision (e.g. confidence/credible interval) and measures of statistical heterogeneity. If comparing groups, describe the direction of the effect. | **☑** P 6 - 7 |
|  | 20c | Present results of all investigations of possible causes of heterogeneity among study results. | **☑** P 5 |
|  | 20d | Present results of all sensitivity analyses conducted to assess the robustness of the synthesized results. | **☑** Not applicable |
| Reporting biases | 21 | Present assessments of risk of bias due to missing results (arising from reporting biases) for each synthesis assessed. | **☑**  Not applicable |
| Certainty of evidence | 22 | Present assessments of certainty (or confidence) in the body of evidence for each outcome assessed. | **☑** Not applicable |
| **DISCUSSION** | | |  |
| Discussion | 23a | Provide a general interpretation of the results in the context of other evidence. | **☑** P 7 - 8 |
|  | 23b | Discuss any limitations of the evidence included in the review. | **☑** P 8 |
|  | 23c | Discuss any limitations of the review processes used. | **☑** P 8 |
|  | 23d | Discuss implications of the results for practice, policy, and future research. | **☑** P 8 |
| **OTHER INFORMATION** | | |  |
| Registration and protocol | 24a | Provide registration information for the review, including register name and registration number, or state that the review was not registered. | **☑** P 4 |
|  | 24b | Indicate where the review protocol can be accessed, or state that a protocol was not prepared. | **☑** P 4 |
|  | 24c | Describe and explain any amendments to information provided at registration or in the protocol. | **☑** P 5 |
| Support | 25 | Describe sources of financial or non-financial support for the review, and the role of the funders or sponsors in the review. | **☑** P 9 |
| Competing interests | 26 | Declare any competing interests of review authors. | **☑** P 9 |
| Availability of data, code and other materials | 27 | Report which of the following are publicly available and where they can be found: template data collection forms; data extracted from included studies; data used for all analyses; analytic code; any other materials used in the review. | **☑** P 9 |

**Supplemental File 2 – List of studies assessed for full-text eligibility**

| **Authors** | **Year** | **Study Title** |
| --- | --- | --- |
| Included studies: 3 | | |
| Silbernagel et al | 2001 | Eccentric overload training for patients with chronic Achilles tendon pain – a randomised controlled study with reliability testing of the evaluation methods |
| Mayer et al | 2007 | Effects of short-term treatment strategies over 4 weeks in Achilles tendinopathy |
| Horstmann et al | 2013 | Whole-Body Vibration Versus Eccentric Training or a Wait-and-See Approach for Chronic Achilles Tendinopathy: A Randomized Clinical Trial |
| No applicable exercise intervention performed: 5 | | |
| Silbernagel et al | 2007 | Continued sports activity, using a pain-monitoring model, during rehabilitation in patients with Achilles tendinopathy: a randomized controlled study |
| Koszalinski et al | 2020 | Trigger point dry needling, manual therapy and exercise versus manual therapy and exercise for the management of Achilles tendinopathy: a feasibility study |
| Malmgaard-Clausen et al | 2021 | No Additive Clinical or Physiological Effects of Short-term Anti-inflammatory Treatment to Physical Rehabilitation in the Early Phase of Human Achilles Tendinopathy: A Randomized Controlled Trial |
| Krogh et al | 2022 | An Isometric and Functionally Based 4-Stage Progressive Loading Program in Achilles Tendinopathy: a 12-Month Pilot Study |
| Chimenti et al | 2023 | The effects of pain science education plus exercise on pain and function in chronic Achilles tendinopathy: a blinded, placebo-controlled, explanatory, randomized trial |
| No applicable outcome parameters: 2 | | |
| Romero-Morales et al | 2018 | Effectiveness of Eccentric Exercise and a Vibration or Cryotherapy Program in Enhancing Rectus Abdominis Muscle Thickness and Inter-Rectus Distance in Patients with Chronic Mid-Portion Achilles Tendinopathy: A Randomized Clinical Trial |
| Romero-Morales et al | 2019 | Ultrasonography effectiveness of the vibration vs cryotherapy added to an eccentric exercise protocol in patients with chronic mid-portion Achilles tendinopathy: A randomised clinical trial |

| **Supplemental File 3 – Characteristics of the interventions in the included studies** | | | | | |
| --- | --- | --- | --- | --- | --- |
| Study (year) | Intervention groups | Intervention | | | Adherence |
|  |  | Total duration,  Intervention modalities | Exercise training movement  (sets x repetitions unless stated) | Frequency |  |
| Silbernagel et al. (2001) (27) | A: Silbernagel protocol  B: CT | 12 weeks  Group A;  Exercise treatment involved in balance,  isometric, concentric/eccentric,  eccentric loadings  Group B;  Exercise treatment involved in concentric/eccentric,  eccentric loadings | Group A;  Week 1:  • Two-legged concentric/eccentric toe raises (3 x 20),  • One leg standing balance (5 x 30 sec)  • Walking on toes or heels (5 x 5 meters)  • Calf muscle stretching with extended and flexed knee (3 x 20 s)  W 2-3: same as week 1 but increase of toe raises;  • Two-legged concentric/eccentric toe-raises (2 x 20)  • One-legged concentric/eccentric toe-raises (3 x 5 to 15 reps by adding 2 reps each day),  • When able to perform 15 reps of concentric/eccentric toe raises: one-legged eccentric toe-raises (10 reps and adding 2 reps each day)  • Stretching of the calf muscles (20 s)  W 4 -12: same as the phase 2 but increase of toe raises;  • Two-legged  concentric/eccentric toe-raises (2 x 20)  • One-legged concentric/eccentric  toe raise on a step (3 x 15 by adding 2 reps each day if tolerated) immediately followed by one-legged eccentric toe-raises on a step (10 reps by adding 2 reps per day if tolerated)  • Quick rebounding toe raises starting  on two-legged to one-legged (3 x 20 to 100 reps)  • Stretching of the calf muscles (20 s)  Group B;  •Concentric/eccentric toe raises (2 x 30 to 3 x 5 reps by adding 2 reps with loads each day)  • When symptoms allowed: one-legged eccentric toe-raises  • Calf muscle stretching with extended and flexed knee (3 x 20 s) | Group A;  Week 1: Three times a day  W 2-3: Two times a day  W 4-12: Once a day for balance and gait exercises, three to four times a week for toe-raises  Group B;  Week 1-12: Three times a day | Not reported  Not reported |
|  |  |  |  |  |  |
| Mayer et al (2007) (26) | A: Physiotherapy intervention (sensory motor training + deep- friction massage + local pulsed ultrasound + ice)  B: Custom insole  C: Control | 4 weeks  Group A;  Exercise treatment involved in balance, stabilization, eccentric, jump loadings  Group B;  Individually fitted insoles provided on the basis of a dynamic plantar pressure distribution measurement  Group C;  Continued normal activities | Group A;  W 1-4:  • Balance and stabilization exercises on a stability pad  • Eccentric heel-drop exercises  • Drop-jumps, and counter-movement jumps  (3 x 15 for every exercise) | Group A;  W 1–4: Two or three times a week  Group B;  W 1-4: Insoles had to be worn for all physical activities  during the treatment phase | Not reported  Not reported  Not reported |
| Horstmann et al (2013) (25) | A: Whole-body vibration training  B: ET  C: Wait-and-see approach | 12 weeks  Group A;  Exercise treatment involved in vibration,  concentric/eccentric loadings  Group B;  Exercise treatment involved in concentric/eccentric loadings  Group C;  Maintained recreational activities | Group A;  Week 1 -12:  • One leg standing on a vibration platform (participants intermittently changed their position between heel rises and heel drops at the edge of the platform until the onset of fatigue)  • Load was increased using vibration frequency (16 to 21 Hz), amplitude (0.5 to 0.8 mm), and exercise time (4 to 7 minutes)  Group B;  Week 1 -12:  • One leg eccentric loadings with straight knee (3 x15)  • Participants performed 4 sets, 15 reps eccentric loadings if no fatigue was occurred with increased load | Group A;  W 1 – 12: Three times a week  Group B;  W 1 – 12: Three times a week | 2.2 training sessions per week (73%)  3.0 training sessions  per week (100%)  Not reported |

**Supplemental File 4 – Author Contributions**

**MyoungHwee Kim** - Conceptualization, Data curation, Formal Analysis, Funding acquisition, Investigation, Methodology, Project administration, Resources, Validation, Visualization, Writing – original draft, Writing – review & editing.

**Wille Martin** - Data curation, Investigation, Methodology, Resources, Validation, Writing – review & editing.

**Andrew Quarmby** - Methodology, Writing – review & editing.

**Josefine Stoll** - Conceptualization, Methodology, Writing – review & editing.

**Tilman Engel** - Conceptualization, Methodology, Writing – review & editing.

**Michael Cassel** - Conceptualization, Methodology, Project administration, Supervision, Writing – review & editing.
